# Breast Vascularization and Its Implication in Breast Reduction and Mastopexy Surgery: Anatomical Study

**DOI:** 10.3390/jpm14050536

**Published:** 2024-05-17

**Authors:** Ainhoa Salas-López, Carolina Morgado-Águila, Carlos López-de-Celis, Jacobo Rodríguez-Sanz, Sara Ortiz-Miguel, Albert Pérez-Bellmunt

**Affiliations:** 1 Hospital Universitario de Bellvitge, L’Hospitalet De Llobregat, 08907 Barcelona, Spain; asalas@bellvitgehospital.cat; 2 Hospital Universitario de Caceres, 10004 Cáceres, Spain; 3 Department of Physiotherapy, Faculty of Medicine and Health Sciences, Universitat International de Catalunya, 08195 Barcelona, Spain; 4 ACTIUM Functional Anatomy Group, Sant Cugat del Vallés, 08195 Barcelona, Spain; jrodriguezs@uic.es (J.R.-S.); sortiz@uic.es (S.O.-M.); aperez@uic.cat (A.P.-B.); 5 Department of Basic Sciences, Faculty of Medicine and Health Sciences, Universitat International de Catalunya, 08195 Barcelona, Spain

**Keywords:** breast, nipple, vascularization, areola complex, breast surgery, breast reduction, Wise pattern

## Abstract

(1) Background: Breast reduction is one of the most frequently performed plastic surgeries in women worldwide. The Wise pattern breast reduction is one of the most frequent skin designs for this surgery. One key point of the surgery is to preserve a well-vascularized NAC by using different surgical pedicles. This study aims to test and update the anatomical knowledge of breast vascularization, the topographic and anatomical basis of the different surgical vascular pedicles, and the differences between the right and left sides. (2) Methods: A descriptive observational anatomical study was carried out on 15 breasts from 10 cryopreserved body donors. A dissection was performed by quadrants to know the affected arteries’ origin in the different patterns. (3) Results: The largest and most frequently dissected internal mammary perforator artery was in the second intercostal space. A total of 44.9% of the dissected perforators are located in the upper inner quadrant, compared to 53.5% in the lower quadrants. (4) Conclusions: The upper inner quadrant alone has the most arterial perforators. In contrast, the sum of the two lower quadrants represents the greatest vascularization of the breast, with a small difference between both.

## 1. Introduction

The breast is a well-vascularized anatomical structure formed by both glandular and fatty tissue. The glandular tissue is under the influence of different hormones, such as oestrogen [1], progesterone [1,2], or prolactin [3,4], so over the years, because of hormonal changes, the mammary gland undergoes volume modifications that condition the overall breast volume and shape. The fatty tissue that conforms to the breast can also increase or decrease according to changes in body weight or other factors. Breast ptosis is in a drop position and it is characterized by an inferior descent of the nipple relative to the breast fold, as well as lower pole skin redundancy [5].

Breast ptosis has been related to hormonal regression, both postpartum and menopause, weight loss, and dermokalasia [6,7]. A gland weight greater than 400g and laxity in internal ligaments can also be considered causes [6,8]. Some authors consider that breastfeeding can cause breast ptosis [9], while other authors consider breast ptosis a consequence of pregnancies and the number of previous pregnancies, besides breastfeeding [6]. Regardless of the consequence, breast ptosis is not only an aesthetic problem but can also affect patients’ self-esteem and self-perception. It has also been related to physical problems such as infection or intertrigo of the submammary fold [10] and cervical and dorsal pail in heavier breasts [8,11].

Breast ptosis has been classically classified into three different stages [12], which consider the nipple position and its relation to the submammary fold. A fourth stage, pseudoptosis, is added to this classification, in which the gland falls from the submammary fold while the nipple stays over this anatomical limit [12]. Some other classifications have been proposed to improve the relationship between the ptosis stage and possible corrective surgery [13]. The suprasternal notch to nipple distance (SSN-N) is an anatomical measurement that can be related to the ptosis stage, since a higher distance could be associated with a more advanced ptosis. However, there is no direct link between them because of the individual factors such as patients’ height or the natural position of the sub mammary fold (SSF), which can vary between patients. The ideal nipple position and SSN-N distance, and the real SSN-N distance may affect the chosen surgical procedure for breast ptosis correction, among other anatomical and anthropometrical measurements.

Mastopexy (or breast lift) is one of the most frequent surgeries worldwide and can correct breast ptosis. Breast reduction is the gold standard surgery to reduce breast volume. Both surgeries can be considered different ends on the same continuum. Both surgeries can lift the mammary gland and reposition the nipple–areola complex (NAC) to an ideal height. Different techniques can be used depending on the ptosis stage and the breast volume (some ptosis can be corrected by mammary augmentation with breast implants). Besides repositioning the gland and the NAC positions, one key point in the surgery is to ensure the vascular supply to the NAC to avoid partial or total necrosis. NAC necrosis has been reported in 1% of mastopexy surgeries [14], while in breast reduction surgeries, NAC necrosis occurs in around 2% [14,15]. Other complications, such as epidermolysis, occur in 5–11% of the surgeries performed [14]. Multiple skin pattern designs can be used to achieve gland lift and skin reductions, the Wise pattern being one of the most frequently used patterns [16,17]. Multiple vascular pedicles can be chosen to ensure the vascular supply to the NAC. Those pedicles can be used with different skin patterns for glandular reduction and breast lift. One of the most commonly used is the superomedial pedicle, based on the internal mammary artery. In the USA, the most frequently used is the inferior pedicle because of its safety and reliability. The double vertical pedicle described by McKissock includes vascularization of both the superomedial and inferior pedicles. Choosing the pedicle must depend on the safest technique for the patient according to their breast characteristics and what they expect from the surgery, as well as the personal preferences of the surgeons [18].

The internal mammary artery, the lateral thoracic artery, and the anterior intercostal arteries are the primary sources of breast vascularization. Other arteries, such as the posterior intercostal artery and superficial thoracic artery, have also been described [18].

This study aims to quantify the number of arterial vessels that can be provided by the pedicles used for this surgery, as well as to describe the main trunks that provide vascularization to the breast and their characteristics in anatomical specimens with breast ptosis. The possible differences in caliber and vascularization between both sides of the same specimen will also be evaluated.

## 2. Materials and Methods

### 2.1. Study Design and Ethics

A descriptive observational anatomical study was carried out at the Universitat Internacional de Catalunya (UIC) anatomy laboratory. Ethical approval was obtained from the institutional ethics committee (CBAS-2023-14). The investigation was performed with a two-step dissection of the breast. The first step was an intraglandular dissection to identify two of the most-used vascular pedicles, and the second step was a deep dissection to locate the main trunks and perforators, and to describe their localization and characteristics. Differences in vascularization between the two sides of the same subject were studied. The study was performed between September 2023 and January 2024.

### 2.2. Anatomical Samples

Female donor bodies with breast ptosis were included. Due to the risk of alterations in vascularization, bodies with previous thoracic surgeries and oncological donors were excluded. The body donors with a history of vascular pathologies or alterations were also excluded.

A total of 15 bodies were cryopreserved until the date of experimentation. Of the total number of bodies in the study, five bodies were excluded:

- Two bodies were excluded due to surgical scars in the thoracic region.
- One body was excluded due to previous breast augmentation surgery with silicone implants.
- Two more bodies were excluded before the study due to prior tests of the dissection procedure technique with intravascular latex injection.

There was a final sample of 10 bodies, of which fifteen breasts were included for the study, discarding five: three breasts were discarded due to the presence of port-a-caths nearby, one due to a pacemaker implanted in the same hemithorax, and one breast due to previous mastectomy surgery. The breasts were studied as independent units. Only the parameters for determining whether there were significant differences between the left and right sides were compared bilaterally.

The mean age at death of the bodies included in the study was 69.2 ± 14.4 years. Frozen carcasses were stored at −20 °C and thawed at room temperature 48 h before the study.

### 2.3. Experimental Procedure

Once the bodies were thawed (48 h before), the study was initiated with the following procedures: (1) macroscopic review of donors and discarding of non-candidate patients; (2) evaluation of the degree of ptosis and design of the surgical pattern; (3) glandular dissection for localization and evaluation of vascularization (step 1 dissection); and (4) deep dissection for localization and evaluation of the main trunks (step 2 dissection).

#### 2.3.1. Assessment of the Degree of Ptosis and Design of the Surgical Pattern

The donor was placed in an upright position, and the degree of breast ptosis was evaluated according to Regnault classification [12]. We measured the suprasternal notch-to-nipple distance (SSN-N) and according to the breast characteristics, the surgical skin pattern for breast reduction–mastopexy for ptosis correction was marked on the skin with a dermographic marker pen. The design of the superomedial pedicle, the inferior pedicle, and the double vertical pedicle were also marked.

#### 2.3.2. Step 1 Dissection

**Figure 1:**
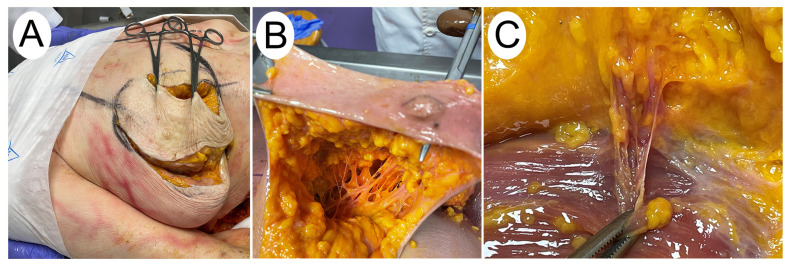
(**A**) Delimitation of the reduction pedicle. (**B**) Intraglandular dissection. (**C**) Internal mammary perforator artery.

The donor was placed in supine position as in a clinical setting. The lower pedicle, modified to include the vertical double pedicle, was dissected according to standard surgical procedure. The skin and subcutaneous adipose tissue of the superficial fascia were removed in layers; a detailed dissection was performed in layers and following anatomical treatment recommendations (Figure 1A). The arteries contained in each pedicle were then dissected. How many arteries belonging to the inferior flap (limiting the count to the upper edge of the areola), the superomedial flap (limiting the count to the lower edge of the areola), as well as those found in both pedicles (in the areola) (Figure 1B,C) were identified. For this procedure, we had the participation of two plastic surgery specialists, who designed the breast reduction–mastopexy skin design and vascular pattern, and two anatomy specialists.

#### 2.3.3. Step 2 Dissection

Once the first step of the dissection was completed, we moved on to the second step. This second phase studied deep vascularization, as well as both perforators and main arterial trunks. Dissection was started from the submammary fold (SMF) in a cranial direction, following the prepectoral plane. The perforators’ location was noted according to the intercostal space they occupied and the mammary quadrant. The ideal position of the NAC was taken as the reference (not the initial position, but the ideal according to the patient’s anthropometric measurements) to establish the breast quadrants.

From the NAC, a horizontal line was drawn parallel to the ground, delimiting the upper and lower quadrants. The breast meridian delimited the inner and outer quadrants, and it is a line that starts at the clavicle 6 cm from the jugular notch and passes through the NAC (because of its ideal position) to the SMF. This way, we located the upper outer quadrant, lateral to the vertical line and superior to the horizontal, and the upper inner quadrant, medial to the vertical line. Below the horizontal line, the two lower quadrants are below the horizontal line, the inner quadrant medial to the vertical line and the outer quadrant lateral to the vertical line. A digital calliper was used to measure the external caliber of the localized perforators, and the internal caliber was measured with a coupler double-end vessel-measuring gauge (GEM749), used in microvascular surgery. However, it was not possible to measure all the perforators found because some had an internal diameter of less than 1 mm.

**Figure 2:**
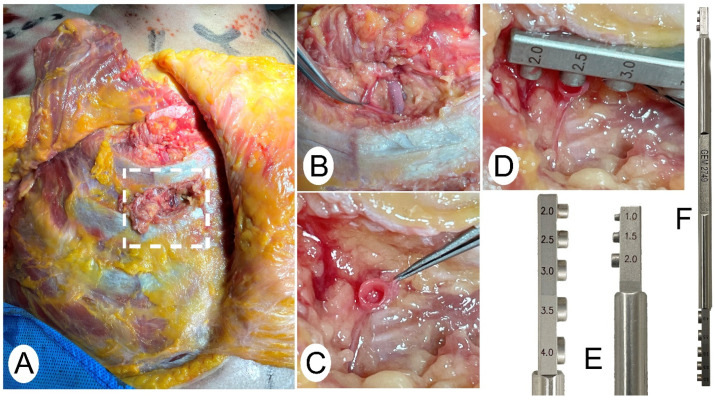
The process of measuring the internal caliber of the internal mammary artery is as follows: (**A**) Dissection of the second intercostal space. (**B**) Dissection of the internal mammary artery. (**C**) Cross-section of the internal mammary artery, proximal end. (**D**) Measurement of the internal diameter using a coupler double-end vessel-measuring gauge. (**E**) Enlarged view of the end with different sizes of vessel measuring coupler gauge. (**F**) Double-ended vessel-measuring coupler gauge.

Once the perforators had been dissected and located, the main vascular trunks were studied. This dissection was assisted by magnifying loupes (Univet. 3x), and microsurgical instruments were used. The internal mammary artery was dissected at the second intercostal space, as shown in Figure 2A,B. A digital caliper was again used for the external measurement, and for the internal measurement, the proximal end of the transverse section of the vessel was taken as a reference (Figure 2C). After dilatation of the artery with microsurgical material, the internal caliber was measured by a coupler measuring gauge, as shown in Figure 2E,F. After measuring the internal mammary artery, the lateral thoracic artery was dissected, repeating the same procedure of external measurement with a digital caliper and measuring the internal caliber at the proximal end of the vessel with a coupler measuring gauge.

### 2.4. Statistical Analysis

IBM SPSS Statistic version 26.0 (Armonk, NY, USA: IBM Corp) software was used for statistical analysis. A descriptive analysis was carried out. The mean and standard deviation were calculated for quantitative variables. Frequencies were calculated for anthropometric and qualitative variables. The Shapiro–Wilk test was used to determine the non-normal distribution of quantitative data. A side-by-side comparative analysis of quantitative variables was performed with the Student’s *t*-test or Mann–Whitney U-test according to the normality assumption. The significance level was set at *p* < 0.05.

## 3. Results

Our sample consisted of 10 female bodies donors with a mean age of 69.2 ± 14.4 years and 15 breasts, 7 right (46.7%) and 8 left (53.3%). Of the breasts, 93.3% had ptosis stage 2 (46.7%) or 3 (46.7%), while only 6.7% (n = 1) had ptosis grade 1. The mean suprasternal notch-to-nipple distance (SSN-N) was 20.8 ± 2.7.

In the first dissection step of the study, we obtained the results of the intraglandular dissection, in which we counted the arteries that would form part of each pedicle. On average, we found almost twice as many arteries in the inferior pedicle (6.1 ± 1.9) than in the superior and superomedial pedicles (3.0 ± 1.1). However, 4.3 ± 3.4 arteries were found in both the superior and inferior pedicles at the same time. (Table 1)

The second dissection step corresponded to the study of deep vascularization, which involves the main trunks and the perforator arteries. Table 1 shows the data on the internal and external caliber of the two main vascular trunks studied: the internal mammary artery and thoracic lateral artery. Concerning the internal mammary, the most frequent location of the perforating branch was the second intercostal space (55.6% of the perforating branches located), while 30.6% were found in the third intercostal space. The internal caliber of the perforators was 1.5 ± 0.7 mm. The location of 80.6% of the perforating branches of the internal mammary were in the upper inner quadrant of the breast (Table 2).

Regarding the intercostal perforating arteries, 90.91% were located in the lower quadrants of the breast, 66.67% in the inner inner quadrant, and 24.24% in the inner outer quadrant. They were mostly located in the fourth and third intercostal spaces. The mean internal caliber of the branches for which information could be obtained was 1.1 ± 0.3 mm. Table 2 details the quadrants and the intercostal space in which the perforator arteries were located.

**Figure 3:**
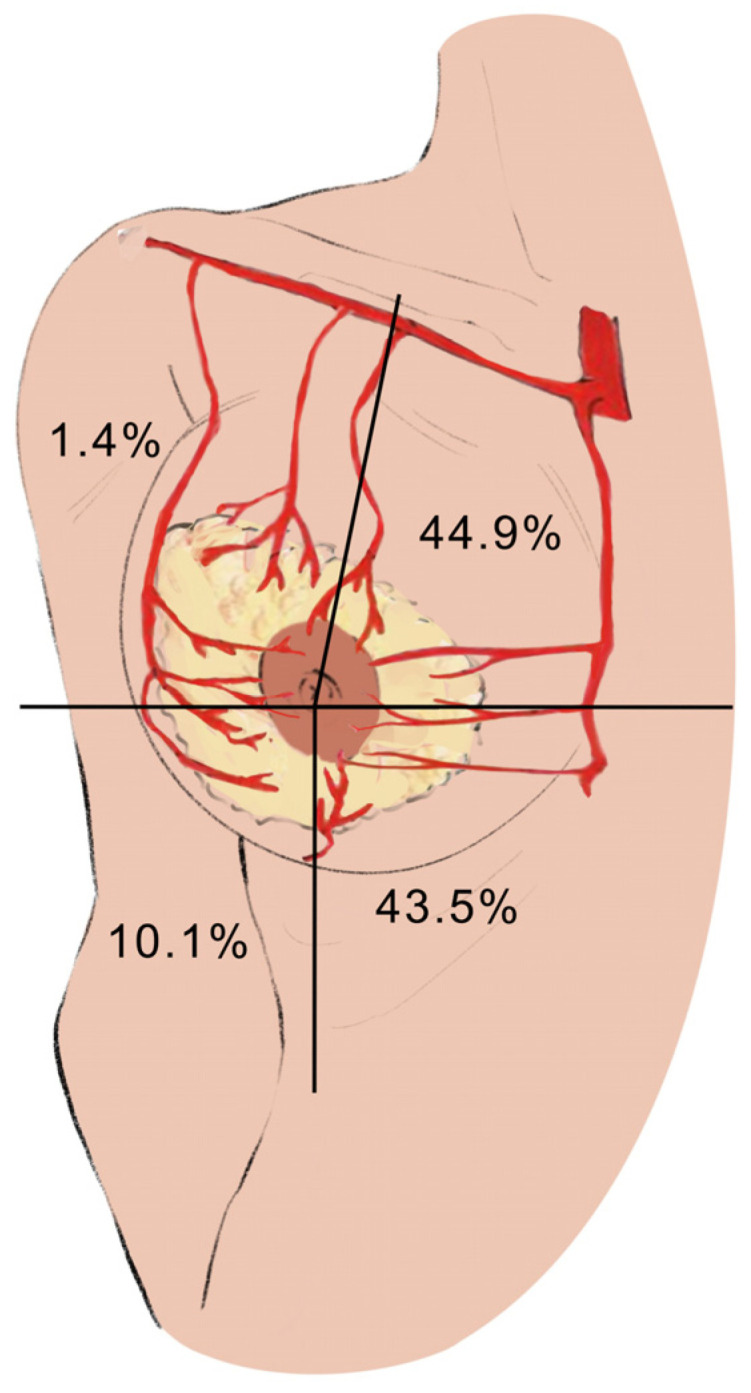
Graphical representation by quadrant and percentage of dissected arteries.

Figure 3 shows the percentage according to the location of the dissected perforating arteries in our sample. As can be seen, the number of perforators in the inner quadrants is much higher than in the outer quadrants. The sum of the two lower quadrants, both the inner and outer, contains more than half of the dissected perforators in our sample. These results are directly related to the vascularization in the different vascular pedicles that can be included in the Wise pattern in breast reduction–mastopexy surgeries.

The comparative data for the right and left sides of specimens in which the breasts were studied bilaterally are shown in Table 3 and Table 4. As shown in Table 3, no statistically significant differences exist in the suprasternal notch-to-nipple distance (SSN-N), and no statistically significant differences were found in the ptosis stage of the studied breasts (Table 4).

When studying the internal mammary artery both internal and external caliber, there are no statistically significant differences between the two sides in our study sample. However, there is a statistically significant difference in the internal caliber of the lateral thoracic artery between the two sides, with the right side being thicker than the left.

## 4. Discussion

This study’s purpose was to provide and update information on intraglandular arterial vascularization and the deep vascularization of the breast. Knowing the breast vascularization can lead to safer breast reduction–mastopexy surgeries and ensure the nipple–areola complex vascular perfusion. In the present sample, a greater intraglandular vascularization was found in the inferior pedicle compared to the superomedial pedicle. In a deeper dissection, we found more artery perforators in the lower quadrants than in the upper quadrants: the upper inner quadrant, specifically.

In this study, the anatomical specimens were dissected under cryopreservation. We dismissed the intravascular latex injection for intraglandular dissection due to difficulty measuring the internal caliber of the perforators and main trunks in the second step dissection. In some other studies, the anatomical dissection was carried out with intravascular injection with lead oxide [18]; some other studies performed an intravascular latex injection [19,20] or used Isovue contrast followed by a dynamic computed tomography (CT) scan [21]. In later studies, the study of breast vascularization was made in vivo by using magnetic resonance imaging (MRI) contrast [14,22]. Indocyanine green is currently used in clinical practice to measure cardiac output, circulating blood volume, and ophthalmic angiography, among other things. Indocyanine green allows intraoperative perfusion of microsurgical flaps to be monitored. Using indocyanine green in the study of breast vascularization could provide information on breast vascularization, without interfering with or altering the anatomy or deep vascularization. [23,24].

The present study is one of the first studies to introduce the coupler double-end vessel-measuring gauge. This device is used in microsurgery to measure the internal vascular caliber in order to to use the assist device in vascular anastomosis. Even though the anastomosis assist device cannot be used in arterial anastomosis, the double-ended vessel-measuring gauge allows the measurement of the internal caliber in arteries and veins. This device only allows internal measurements of vessels larger than 1 mm and, after that, in 0.5 mm intervals.

In our study of intraglandular vascularization in our samples, the arteries located in the inferior pedicle (10.4 ± 5.0) were superior to those in the superomedial pedicle (7.3 ± 3.8). In both cases, the arteries located in the NAC would be included in the dissection of both pedicles. In the case of donors lacking intra-arterial pressure, the data obtained may have been underestimated compared to in vivo patients. The value may have been underestimated even though the assessment was made by two anatomical experts and a plastic surgeon familiar with the technique.

Our study focuses on the internal mammary artery, the lateral thoracic artery, and the perforators of the anterior branch of the intercostal artery, which are the main vascular trunks for breast vascularization [25]. There are other vessels whose contribution is less, which have not been evaluated in this study, such as the perforators of the posterior branch of the intercostal artery and direct branches of the axillary artery [18,20,21].

Regarding the internal mammary artery, we observed that the most constant and most frequently found perforator in the dissection was located in the second intercostal space (55.6%), followed by the perforators located in the third intercostal space with 30.6%. This result agrees with other studies, in which the most frequently located perforators of the internal mammary artery were located between the first and third intercostal space [26], especially in the second intercostal space [19,27]. This second perforator has also been found to vascularize a larger cutaneous territory [27,28].

In our sample, the internal mammary artery’s internal caliber was 2.7 ± 0.3 mm. In comparison, the external caliber of the vessel was 3.5 ± 0.8 mm, a value similar to that presented by other authors such as [19], in which the diameter of the internal mammary artery was 3.6 mm. Regarding the caliber of the perforators of the internal mammary artery, the mean in our sample was 1.5 ± 0.7, a value very similar to other studies that have presented a range between 1.6 mm and 1.8 mm [18,27].

Our lateral thoracic artery had an external caliber of 1.5 ± 0.7 mm, a value very similar to that found by other authors [18] where the external caliber was 1.5 ± 0.3 mm.

Regarding the perforators of the anterior branch of the intercostal arteries, the most frequently found were located in the fourth intercostal space (45.5%), followed by the third (23.5%), and fifth (17.6%) intercostal space. These results are in agreement with other studies [18,28]. Other studies [16] relate these fourth and fifth rib perforators to a more direct trajectory to the NAC due to embryology where, prior to development, the nipple–areola complex is primarily located in that position.

We studied possible differences in the breasts of the same subject. Neither the breast ptosis stage nor the suprasternal notch-to-nipple distance showed statistically significant differences. We did not find statistically significant differences in the internal and external caliber of the internal mammary artery according to laterality. Other studies have shown [29] differences between the two sides of the internal mammary veins, with the right vein found to have a larger internal caliber than the left. However, we did find a statistically significant difference in the internal thoracic artery’s internal caliber, the right side being larger than the left one.

This study exclusively evaluated arterial vascularization without dissection and the state of venous drainage. Venous congestion is one of the causes of complications in breast reduction–mastopexy surgery, as it can lead to suffering and partial or total necrosis of the NAC [30]. As our study cannot draw any conclusions, further studies focusing on the state of the breast’s venous system are necessary.

A total of 80.6% of the perforators of the internal mammary artery were located in the upper inner quadrant of the breast, the main vascular supply to the superior and superomedial pedicle. Concerning the total vascularization of the breast, the inner quadrant represents 44.9% of the vascularization. A total of 53.6% of the vascularization was located in the lower quadrants, both inner and outer. The lower quadrants are the ones that provide the vascularization to the lower pedicle in breast reduction–mastopexy surgeries and are derived mainly from the perforators of the anterior branch of the intercostal artery. Our sample does not show significant differences between the upper inner and lower quadrants, so we cannot choose a more clinically reliable pedicle. Improving knowledge of breast vascularization allows for more precise and reliable surgical techniques, both in mastopexy–reduction surgeries with a Wise pattern or other patterns, as well as in other breast surgeries, including breast-conserving oncological surgeries and their reconstructive options.

### Limitations

The main limitation of the study is the small sample size. On the one hand, due to the legislation in force in our country, according to which the clinical data of the donor cannot be revealed, the clinical history is unknown, including smoking, disease in the breast tissue, or a history of arteriopathies, which could condition the results obtained and make it difficult to apply the results obtained to the general population. On the other hand, the mean age of the specimens is higher than the mean age of most patients undergoing mastopexy–reduction surgery. Also, we cannot guarantee that these data are the same in other races, which were not present in our sample.

The most complex cases on a clinical level regarding the vascularization of the NAC are those with a suprasternal notch-to-nipple distance of 30 cm and above [30], especially those above 40 cm [17]. The SSN-N mean in our studied sample was 20.8 ± 2.7, so our results must be carefully extrapolated to extreme cases.

In the evaluation of the intraglandular arteries of the breast, despite having been analyzed by two anatomy experts and a plastic surgeon, the lack of intravascular arterial pressure makes assessment and quantification difficult, so the impact of the data obtained in the population is limited. New studies studying intraglandular vascularization with pressure could provide new data. The results should, therefore, be viewed with some caution.

## 5. Conclusions

The upper inner quadrant, which provides vascularization to the superior and superomedial pedicle in breast reduction–mastopexy surgeries, has the highest number of perforators. The sum of the two lower quadrants, both internal and external, which represent the vascular contribution of the lower pedicle, accounts for more than 50% of the breast’s vascularization. In terms of vascularization, there are no major differences in the different vascular pedicles that can be included in a surgical procedure in a Wise pattern breast reduction–mastopexy.

## Figures and Tables

**Table 1:** Intraglandular and main trunks study.

	Mean ± SD
Superior pedicle	3.0 ± 1.1
Common pedicle	4.3 ± 3.4
Inferior pedicle	6.1 ± 1.9
External caliber internal mammary artery	3.5 ± 0.8 mm
Internal caliber internal mammary artery	2.7 ± 0.3 mm
External caliber thoracic lateral artery	1.5 ± 0.6 mm
Internal caliber thoracic lateral artery	1.8 ± 0.4 mm

Abbreviations: SD, standard deviation.

**Table 2:** Perforator arteries information.

	Internal Mammary Artery (n = 36)	Intercostal Perforator Arteries (n = 33)
	Mean ± SD or n (%)	Mean ± SD or n (%)
Upper outer quadrant	-	1 (3.03%)
Upper inner quadrant	29 (80.6%)	2 (6.06%)
Lower inner quadrant	7 (19.4%)	22 (66.67%)
Lower outer quadrant	-	8 (24.24%)
First intercostal space	2 (5.6%)	-
Second intercostal space	20 (55.6%)	-
Third intercostal space	11 (30.6%)	12 (36.36%)
Fourth intercostal space	3 (8.3%)	15 (45.46%)
Fifth intercostal space	-	4 (12.12%)
Sixth intercostal space	-	2 (6.06%)
External caliber	0.9 ± 0.8	1.5 ± 0.6
Internal caliber	1.5 ± 0.7	1.1 ± 0.3

Abbreviations: SD. standard deviation; n. number.

**Table 3:** Comparison between both sides.

	Right	Left	
	Mean ± SD	Mean ± SD	*p*-Value
SSN-N	22.3 ± 2.3	21.4 ± 2.6	0.202
Internal mammary A. Internal caliber	3.5 ± 0.3	3.2 ± 0.7	0.756
Internal mammary A. Internal caliber	2.7 ± 0.3	2.7 ± 0.3	1.000
Lateral thoracic A. External caliber	1.5 ± 0.7	1.8 ± 0.5	0.251
Lateral thoracic A. Internal caliber	2.0 ± 0.4	1.5 ± 0.0	0.017

Abbreviations: SD, standard deviation; SSN-N, suprasternal notch-to-nipple distance.

**Table 4:** Comparison of ptosis stage.

Ptosis Stage	Left Side	Right Side	Total
Stage 1	3	2	5
Stage 2	2	2	4
Stage 3	0	1	1

## Data Availability

The data presented in this study are available on request from the corresponding author.
